# Therapeutic Endoscopic Ultrasonography

**DOI:** 10.1155/2013/390821

**Published:** 2013-09-25

**Authors:** Everson L. A. Artifon, Marc Giovannini, Siyu Sun, Juan J. Vila

**Affiliations:** ^1^Department of Surgery, University of Sao Paulo, Rua Guimaraes Passos 260, Vila Mariana, 04107-030 Sao Paulo, SP, Brazil; ^2^Gastroenterology and Endoscopy Department, Paoli-Calmettes Institute, 232 Boulevard Sainte Marguerite, 13273 Marseille Cedex 9, France; ^3^Endoscopy Center, Shengjing Hospital of China Medical University, 36 Sanhao Street, Shenyang, China; ^4^Endoscopy Unit, Gastroenterology Department, Complejo Hospitalario de Navarra, Irunlarrea, 331008 Pamplona, Spain

The development of linear sectorial array EUS scopes in the early 1990s brought a new approach to diagnostic and therapeutic dimensions on EUS capabilities, opening the possibility to perform punction over direct EUS guidance. Endoscopic ultrasound (EUS) has evolved from a purely diagnostic imaging modality to an interventional procedure that provides a minimally invasive alternative to interventional radiologic and surgical techniques. This evolution was catalyzed by the introduction of linear echoendoscopes that provide continuous imaging and observation of needles and by therapeutic devices that pass through large-caliber working channels. The spectrum of EUS-guided interventions is today quite large including drainage of the pancreas, gallbladder, and other fluid collections; access to the pancreatic and biliary systems; celiac plexus interventions; vascular and ablative therapies.

Some new indications for interventional and therapeutic endoscopic procedures performed under EUS control have been developed in areas that have been purely surgical for many years. Indications, procedures, and related tools for EUS-guided endosurgery are described, all of which are experimental but may open a new corridor for endoscopists to enter a variety of transluminal procedures in real time without soiling the peritoneal or mediastinal cavity.

In this current issue, the readers can have emerging technologies applied to the endoluminal interventions made by using EUS view and another endoscopic resources. On the other hand, the procedures in the therapeutic EUS give us the possibility of having new access through the gastrointestinal wall to closed organs.

Beside new EUS-guided therapies, other authors in this collection have made a thorough review of the therapeutic capabilities of EUS. The application of EUS-guided therapy is especially appealing in oncological diseases, offering accurate and effective palliative treatment. This is possible mainly due to two EUS characteristics which are: firstly, its ability to deliver direct treatment to lesions unreachable by other means and secondly, the minimal invasiveness of EUS which allows outstanding results with low complication rate.

EUS-guided vascular therapy is a new promising field, because the variety of vascular pathology and the proximity of the vascular structures to the GI tract walls call for EUS-guided vascular procedures. Some reports have suggested that EUS-guided vascular procedure might offer benefits for refractory bleeding, and further development of this technique could potentially replace the interventional radiology in the abdominal vessels. A paper in this issue describes the literature review about EUS-guided vascular procedures, such as EUS-guided management of nonvariceal upper GI bleeding, and EUS-guided management of pseudoaneurysms, giving the reader a quick overview of the current state of research of EUS-guided vascular interventions. 

Pancreatic cancer is a significant cause of morbidity and mortality. Current therapies, however, are of limited benefit in most patients. Recently, several studies have shown initial success with injection of various medications and therapies into pancreatic tumors under the EUS guidance. This issue carries articles on EUS-guided injection of AdV-tk into the pancreatic tumor, which provide the ability to treat pancreatic cancer in a relatively minimally invasive manner, with a very low incidence of procedural-related complications. The latest cutting-edge technique might provide hope in treating the lethal disease in the foreseeable future.

In the articles included in this issue, the reader can find new endoscopic approaches by means of EUS to clinical problems or even complications derived from endoscopic maneuvers. These new technical solutions represent one step ahead in therapeutic endosonography. The clinical benefit and safety profile of some of these approaches must be confirmed in new studies, but they open new fields for clinical and experimental research.

The minimal invasive concept is an appropriate nomination to the therapeutic procedures that have been made by endoscopy. The endoscopic ultrasound offers to the endoscopy area a new technological eye to reach nonanatomic trajects creating fistulas or determining cytoreduction of masses. Otherwise, therapeutic endoscopic ultrasound or T-EUS is a new tool to those patients who are not operative candidates and in which we can offer a high quality of life (QOL) during a short survival.

*Everson L. A. Artifon* *Everson L. A. Artifon* *Marc Giovannini* *Marc Giovannini* *Siyu Sun* *Siyu Sun* *Juan J. Vila* *Juan J. Vila*

